# Advanced therapy medicinal products in China: Regulation and development

**DOI:** 10.1002/mco2.251

**Published:** 2023-04-26

**Authors:** Jiaqi Lu, Longchang Xu, Wei Wei, Wu He

**Affiliations:** ^1^ Center for Drug Evaluation National Medical Products Administration Beijing China

**Keywords:** advanced therapy medicinal product (ATMP), gene therapy products, immune cell products, investigational new drug (IND), stem cell products

## Abstract

Advanced therapy medicinal products (ATMPs) have shown dramatic efficacy in addressing serious diseases over the past decade. With the acceleration and deepening of China's drug regulatory reforms, the country sees a continuous introduction of policies that encourage drug innovation. The capacity and efficiency of the Center for Drug Evaluation (CDE), National Medical Products Administration have significantly improved, where substantial resources have been allocated to ATMPs with major innovations and outstanding clinical values that satisfy urgent clinical needs. These changes have greatly stimulated the research and development of biological products in China, ushering in a period of explosive growth in the number of investigational new drug (IND) applications of ATMPs. Here, we described China's ATMP regulatory framework and analyzed data on IND applications for ATMPs submitted to CDE. The data show that China's ATMP industry is expanding dramatically, but lagging behind in terms of the innovative targets and the coverage of indications. However, in recent years, the diversity of product types, targets, and indications is growing. We discussed challenges and opportunities in ATMP regulation. Risk‐based regulation and cross‐discipline collaborations are encouraged to promote more ATMPs toward market authorization in China.

## 1 INTRODUCTION

Advanced therapy medicinal products (ATMPs) are medicines based on genes and cells, including gene therapy products, cell therapy medicinal products, tissue engineered products and other types of products, which are characterized by complex biological features and manufacturing processes.^1^ Generally, ATMPs are used to treat specific diseases with limited effective therapeutic methods, which have become a hot spot in the field of biopharmaceutical research. Due to the unique mechanism of action and biological functions of ATMPs, the therapeutic advantages of ATMPs in some refractory diseases have been gradually recognized, which could potentially cure specific types of diseases and thus targeting the unmet medical needs.^2^

Up to August, 2022, there are 8 chimeric antigen receptor‐T cell (CAR‐T) products and 7 Adeno‐Associate Virus (AAV) products approved on market globally, representing the majority of marketed ATMPs worldwide. Multiple ATMPs initiated pivotal clinical study and hopefully more CAR‐T and other types of products will be approved in the next decade.^3^ In China, National Medical Products Administration (NMPA) regulates ATMP as “innovative biological products” as defined in the Provisions for Drug Registration (NMPA Order No.27, 2020). In 2021, NMPA approved 2 CAR‐T products on market, namely Axicabtagene Ciloleucel Injection and Relmacabtagene Autoleucel Injection. Their indications include relapse/refractory diffuse large B cell lumphoma and follicular lymphoma. By the end of 2022, the new drug application (NDA) filings of 4 CAR‐T products have been accomplished and more ATMPs are predicted to submit NDA in the next following years.

Different from traditional biologicals, ATMPs have brought many new challenges to drug development and regulation in terms of development strategies, technical guidances and regulatory policies. At present, regulatory authorities in various countries and regions have issued many supportive policies and guidances. Within European Medicines Agency and US Food and Drug Administration (US FDA), expedited regulatory pathways were created that could possibly benefit ATMP companies, such as Regenerative Medicine Advanced Therapy in US and Priority Medicines scheme (PRIME) in EU.^4^, ^5^, ^6^ New meeting modality has been created to improve the effective communications between regulators and stakeholders, such as the Initial Targeted Engagement for Regulatory Advice on CBER Products (INTERACT) in US FDA CBER. And many drug regulatory agencies worldwide have issued numerous product‐specific technical guidances, describing the regulators’ opinion and recommendations on ATMP development.^7^

Here we introduce the current ATMP development status and regulatory scenario in China, which mainly focus on the optimization of drug review process and the analysis of investigational new drug (IND) applications of ATMPs. And, we discuss how NMPA improves the regulatory framework to address the challenges and accelerate ATMPs toward clinic.

## 2 THE REVIEW PROCESS AND REGULATORY FRAMEWORK OF ATMPS IN CHINA

With the deepening of regulatory reforms since 2015, the optimization of drug review process with multiple expedited programs cleared many obstacles hindering the development of ATMPs.

### 2.1 The expedited regulatory programs speed up the approval of ATMPs

**FIGURE 1:**
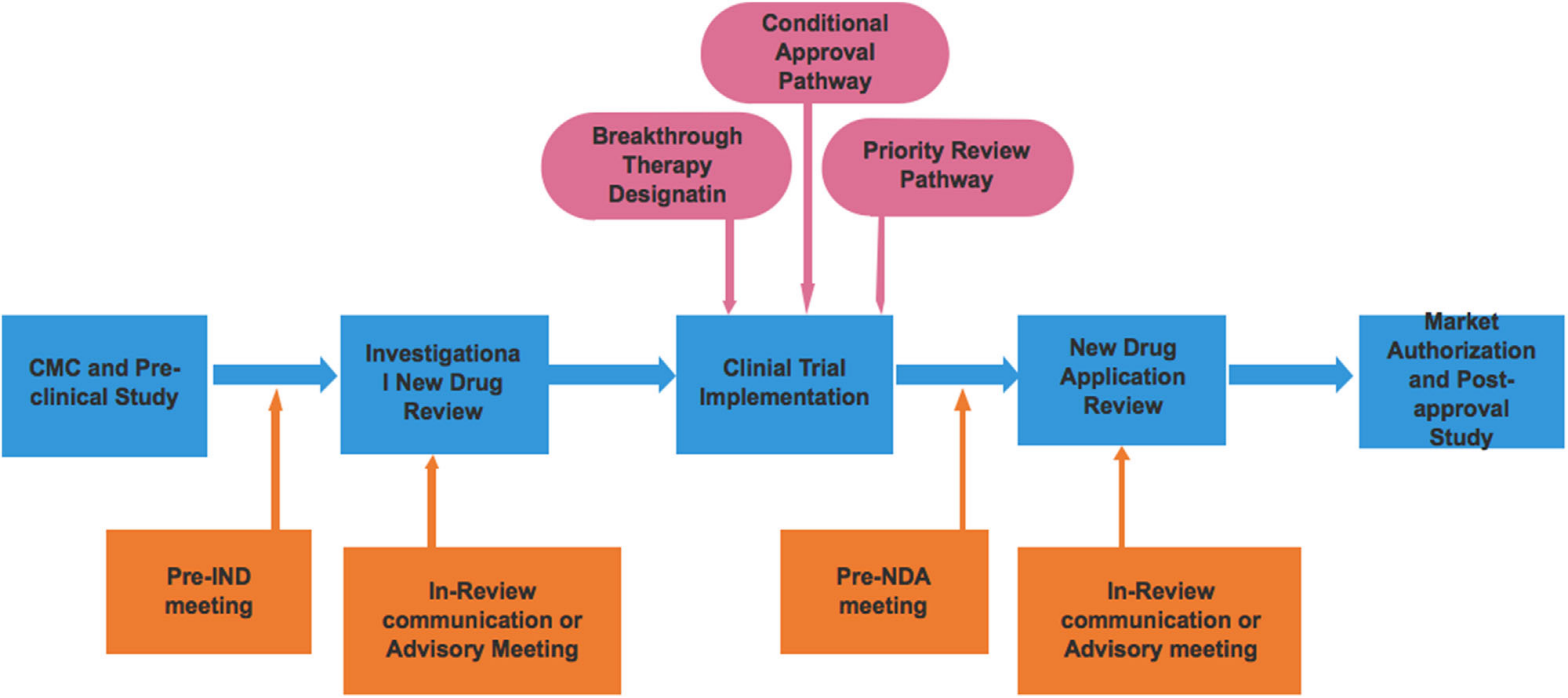
The workflow of Advanced therapy medicinal product (ATMP) review process with expedited programs and formal regulatory meetings. The ATMP development and review process include chemistry, manufacturing and control (CMC) and preclinical study, investigational new drug (IND) review, clinical trial implementation, new drug application (NDA) review, market authorization and post‐approval study. Priority review pathway, breakthrough therapy designation and conditional approval pathway are three expedited programs that are commonly used in ATMP regulation. The formal regulatory meetings include pre‐IND meeting and pre‐NDA meeting, and in‐review communication or advisory meeting may be held if necessary.

To encourage innovative drug development, NMPA accelerates review and approval of ATMPs through multiple expedited programs such as Priority Review Pathway, Breakthrough Therapy Designation, Conditional Approval Pathway (Figure 1). These programs remarkably reduced the time for NDA approval by prioritizing inspection, rolling review, more frequent meetings, etc. Up to November, 2022, a total of 6 CAR‐T products were granted Priority Review Pathway, eight CAR‐T products and two AAV products were granted Breakthrough Therapy Designation during the clinical trial stage, of which two CAR‐T products were conditionally approved on market in 2021.

### 2.2 The drug review process is improved

The Provisions for Drug Registration (NMPA Order No.27, 2020) adjusted drug review process and further shortened the review time.^8^ NDA review should be finished within 200 days of the receipt of the original submission, and 130 days for ATMPs granted Priority Review. Inspection and registration testing procedures were set in parallel with review after 2020, instead of the review‐inspection‐testing mode before 2020. In July, 2018, the NMPA introduced an implied license system for IND approval (number: 50, 2018, NMPA), which limits IND review time from 90 days to 60 days.

### 2.3 Effective meetings facilitate communications

Since 2018, pre‐IND meeting is suggested prior to IND submission to determine whether the data support clinical trial (Figure 1). Large numbers of ATMP pre‐IND and pre‐pre‐IND meetings have been applied, and drug reviewers have been involved in the early developmental stage of ATMPs. The data in ATMP IND files were assessed, and reviewer's feedback help stakeholders improve the master files, resulting in the markedly decrease of IND rejection rate. Meanwhile, advisory committee meetings have been held during review process if needed, to reach convergence between scientists, stakeholders, and regulators (Figure 1).

### 2.4 The regulatory framework of ATMPs is developed

In addition to the existing guidance on biological products, Center for Drug Evaluation (CDE) has issued a series of technical guidance documents describing current thinking on the development of ATMPs. These guidance documents clarified the definitions of each category of ATMPs in China and articulated the regulatory considerations of chemistry, manufacturing and control (CMC), nonclinical and clinical research with regard to the products ranging from immune cell therapy, in vivo gene therapy, ex vivo gene therapy, oncolytic viruses to gene editing and stem cell products (Table 1). Risk‐based compliance is recommended, and communication with regulators is encouraged in these documents.

**TABLE 1:** Guidance documents describing China's Advanced therapy medicinal products (ATMPs) regulatory framework.

Subjects	Document	Summary	Key point
**General**	Guidance for research and evaluation of cellular therapy products^9^	Provides the scope of cellular therapy products. Describes the general technical requirements of CMC, nonclinical, and clinical.	Identify the types of cellular therapy products which can be regulated as “biological products.”
	Guidance for research and quality control of human gene therapy products^10^	Clarifies the definition and categories of gene therapy products, in vivo products and ex vivo products. Describes the general technical requirements of CMC, nonclinical, and clinical.	Risk assessments are essential to ensure the safety of gene therapy products. Regulatory flexibility is recommended.
	Guidance for research and quality control of human somatic cell therapy products^11^	Provides the definition and categories of somatic cell therapy products. Describes the general technical requirements of CMC, nonclinical, and clinical.	Clarifies the technical requirements of raw materials, including human serum and human blood derivatives.
**CMC**	Guidance for CMC research and evaluation of in vivo gene therapy products^12^	Provides the categories of in vivo gene therapy products. Describes the detailed CMC technical requirements, including raw materials, manufacture, quality control, etc.	Indicates the risk‐based CMC study strategy. Illustrate the product‐specific technical requirements, including vector‐based products, nucleic acid products, etc.
	Guidance for CMC research and evaluation of gene manipulation system used to manufacture ex vivo gene therapy products^13^	Provides the categories of gene manipulation systems used to make ex vivo gene therapy products. Describes the detailed CMC technical requirements. Clarifies the risk‐based requirements for the gene editing tools in different using scenarios.	Introduces various types of gene editing tools, including the viral vectors, nonviral vectors and nucleic acids to make CAR‐T and iPSC derivatives.
	Guidance for CMC research and evaluation of immune cell therapy products^14^	Provides the scope of immune cell therapy products. Describes the detailed CMC technical requirements, including raw materials, manufacture, quality control, etc.	Clarifies the regulation of donor cell collection procedures. Illustrates the requirements for production capacity changes.
**Nonclinical**	Guidance for nonclinical research and evaluation of gene therapy products^15^	Clarifies the basic principle of nonclinical study design and implement regarding gene therapy products.	Illustrates how to select proper animal model, the possibility to use of organoids instead of animal model, and the technical points to perform “proof‐of‐concept” study.
	Guidance for nonclinical research and evaluation of gene‐modified cellular products^16^	Describes the study design of nonclinical research specifically to gene‐edited cellular products, including vector‐modified cellular products and CRISPR‐Cas edited cellular products, etc.	Clarifies the technical requirements of tumorigenic/carcinogenic studies.
**Clinical**	Guidance for clinical trial research of immune cell products^17^	Describes the study design of clinical research specifically to immune cell products.	Describes the toxicity and management of CAR‐T products.
	Guidance for long‐term surveillance of gene therapy products^18^	Describes the clinical study design in the follow‐up of patients administered with gene therapy products	Introduces the specific considerations of testing design for integrating vectors.
	Guidance for clinical trial design of oncolytic virus‐based products^19^	Describes the clinical study design of oncolytic virus‐based products, including administrative routes, dosing and clinical endpoint selection, etc.	Describes the design and analysis of shedding studies for oncolytic products.

Abbreviations: CMC, chemistry, manufacturing and control; CAR‐T, chimeric antigen receptor‐T cell; PSC, pluripotent stem cell.

### 2.5 International collaboration promotes regulatory convergence

China's joining the International Conference on Harmonization (ICH) in 2017 paved a way for international collaboration in ATMP regulation. In 2020 ICH management committee meeting, NMPA proposed two reflection papers on the regulation of stem cell‐based products and gene therapy products. Now CDE experts are enrolled in the ICH discussion of viral safety evaluation (ICH Q5A) and nonclinical biodistribution considerations (ICH S12) for gene therapy products.

## 3 ATMP IND APPLICATIONS IN CHINA

### 3.1 ATMP IND application

**FIGURE 2:**
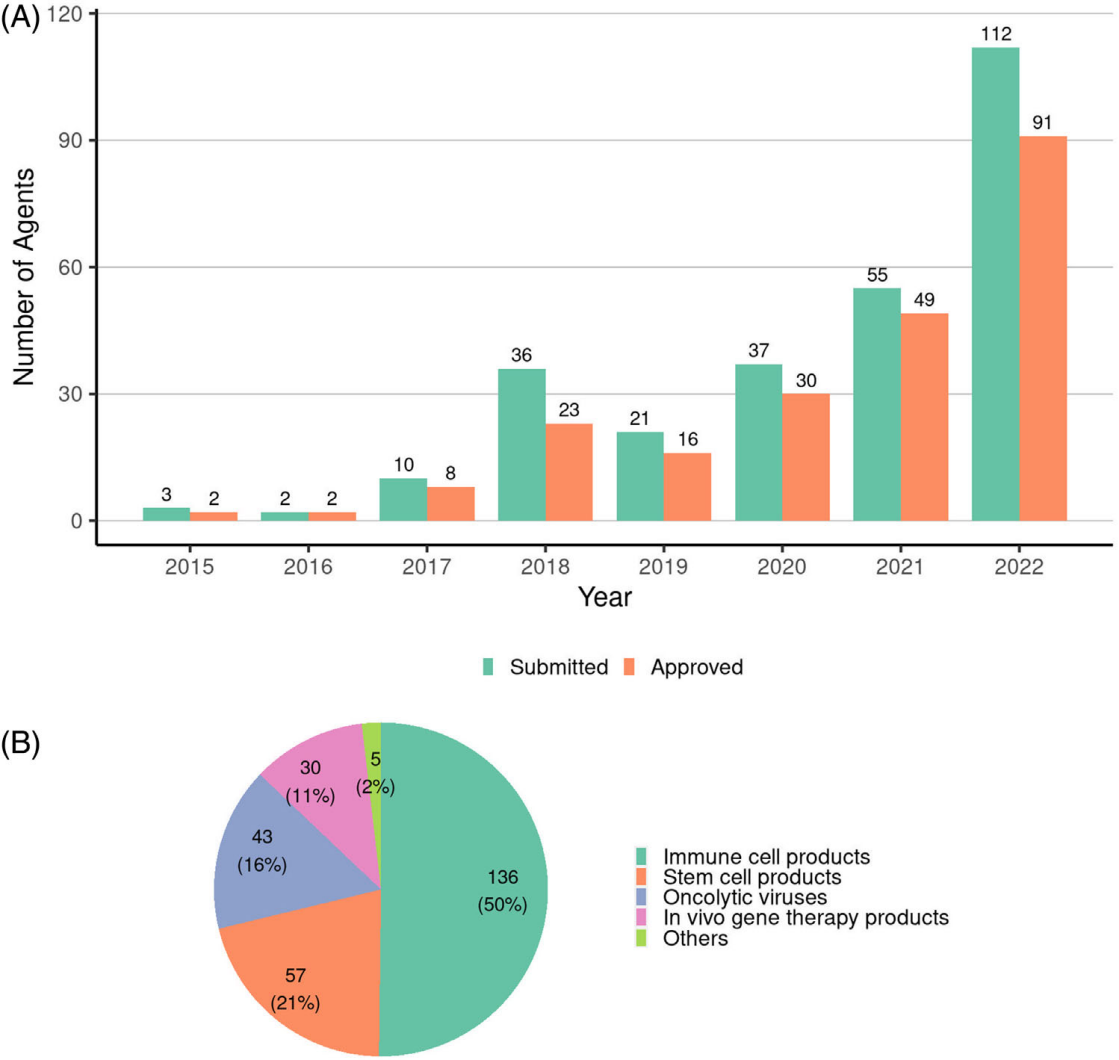
Annual numbers of submitted investigational new drug (IND) applications for advanced therapy medicinal products (ATMPs) in China. (A) Annual numbers of submitted ATMP IND applications (blue bars) and approved applications (red bars) in the year of first submission from 2015 to November, 2022. (B) The submitted IND applications were classified into five main groups, immune cell products, stem cell products, oncolytic viruses, in vivo gene therapy products, and other products. The number and percentage of each group from 2017 to November, 2022 were presented. COVID‐19‐related ATMPs were excluded.

The data on IND applications for ATMPs that were submitted to NMPA from January 1, 2002 to November 30, 2022 were collected from CDE database of NMPA's Registration and Information Disclosure Platform for Drug (https://www.nmpa.gov.cn/yaopin/index.html). COVID‐19‐related ATMPs were excluded. In the past decade, the numbers of ATMP IND applications have increased significantly. Various types of ATMPs were submitted for clinical trials, including stem cell products, immune cell products, oncolytic viruses, etc. According to unpublished data of CDE, 48 INDs of ATMPs were submitted from 2002 to 2016, mostly oncolytic viruses, mesenchymal stem cell products and cytokine‐induced killer cell products. Owing to the publishing of *Guidance for Research and Evaluation of Cellular Therapy Products* consistent with the market authorization of first two CAR‐T products worldwide in 2017, large numbers of CAR‐T products initiated submission in China in 2017 and 2018, and the number of ATMP IND applications increased dramatically in 2018 (Figure 2A). From 2017 to November, 2022, CDE received a total of 271 IND applications of ATMPs (counted as the number of products, not as catalog numbers because one product with different indications may have more than 2 catalog numbers), in which 97.3% of the first IND applications came from local pharmaceutical companies. The number of ATMP IND applications increased from approximately 1.5 per year during the years 2002−2012 to approximately 55 in 2021 (a new high over the past 19 years), with an average annual growth rate of 57%.

Of the 271 IND applications from 2017 to November, 2022, 136 were immune cell products and 57 were stem cell products, representing the majority of ATMPs (Figure 2B). In addition, oncolytic virus products, in vivo gene therapy products, and other personalized treatment products, etc. have also been submitted and approved. Of the 271 ATMP IND reviews, 217 were approved and 54 were rejected. The reasons for rejection mainly include a lack of essential data, safety issues in the raw materials, failure to properly perform safety testing such as replication‐competent lentivirus test, and that incomparability between toxicological batches and clinical trial batches.

### 3.2 Product types, targets, and indications

**FIGURE 3:**
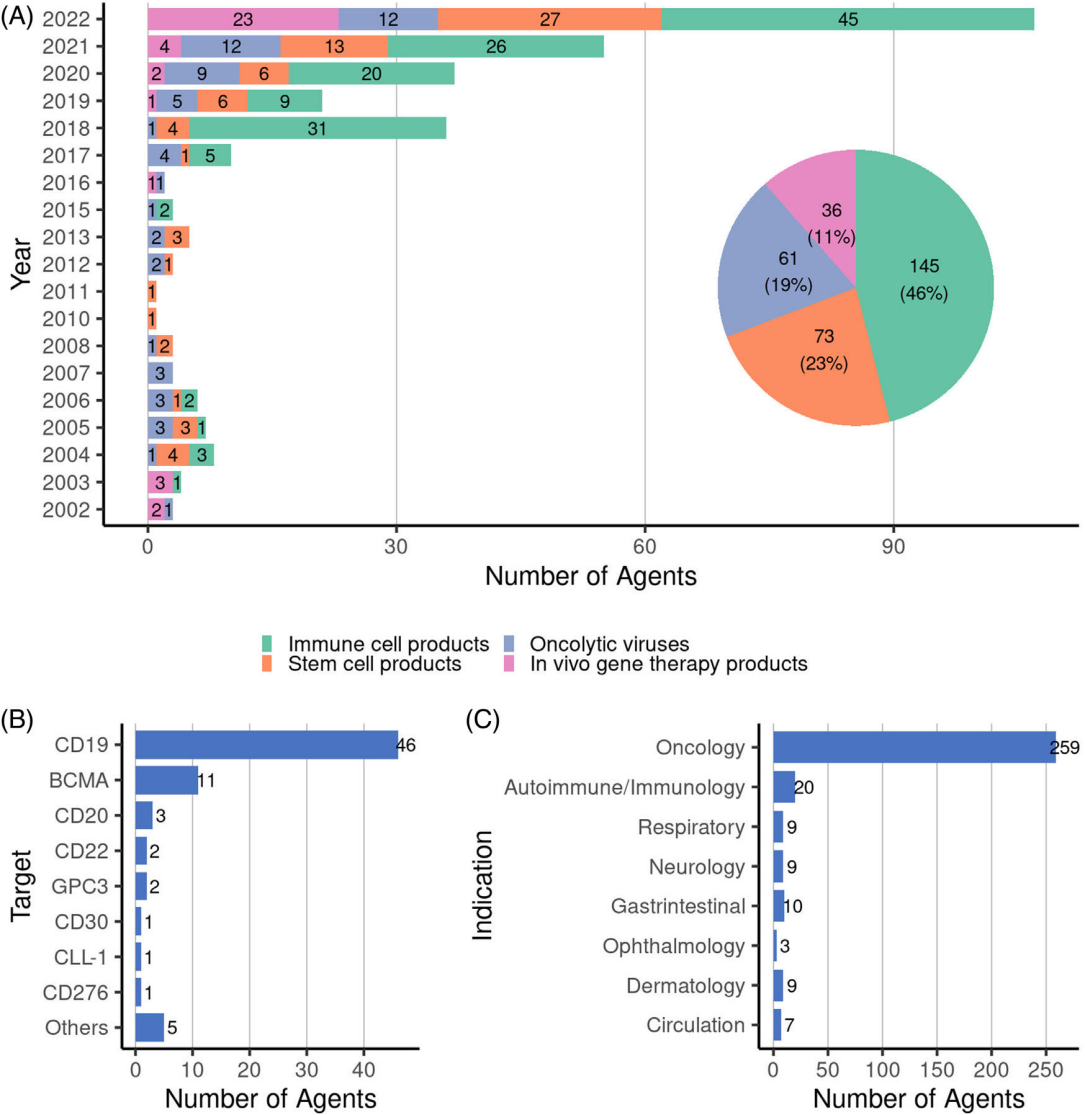
Developmental status of the Advanced therapy medicinal products (ATMPs). (A) ATMP investigational new drugs (INDs) submitted per year from 2002 to 2022 were classified into four groups including immune cell products, stem cell products, oncolytic viruses and in vivo gene therapy product. And, the percentage of each group was presented in pie chart. (B) Drug targets were identified with regard to chimeric antigen receptor‐T cell (CAR‐T) products submitted from 2017 to November, 2022. The products target either a single target or multiple targets. (C) The distribution of indications of all submitted ATMP INDs from 2017 to November, 2022. Biomarker‐based indications were excluded.

Overall, the diversity of product types, targets, and indications is growing. From 2002 to 2016, most ATMPs submitted were oncolytic viruses and cell products without genetic manipulation procedures. Since 2017, as a rapid follow‐up of approved CAR‐T products in the world, various types of cellular products were submitted, such as CAR‐T, TCR‐T, TIL, CRISPR‐Cas9‐modified cell products. The types of ATMPs approved for IND have gradually diversified from 2019, when CAR‐T products with multiantigen targeting, stem cell products and AAV products became popular (Figure 3A). Among them CAR‐T represented the largest number of ATMPs submitted. As of November 2022, 46 CD19 CAR‐T INDs have been submitted in China. The number of novel types of CAR‐T also increases, including universal CAR‐T, fast‐made CAR‐T and nonviral vector‐modified CAR‐T products, etc.^20^

In terms of the targets of CAR‐T products, 79.2% of CAR‐T products focused on two targets, CD19 and BCMA (Figure 3B). Other targets of the minority of products include CD20, CD22 and solid tumor targets such as GPC3. In stem cell field, mesenchymal stem cell products accounted for 88% of the stem cell products from 2004−2019. In 2021, this proportion has reduced to 76%. Notably, the majority of ATMP INDs from 2017 to November 2022 had repeated targets, similar cell types and indications (Figure 3B,C). Oncology diseases are the main indication for all submitted ATMP INDs. Most CAR‐T products were indicated to treat hematological cancers, while four solid tumor CAR‐T products have been approved in clinical trial.

Seventy‐three INDs of stem cell products have been submitted during 2004−2022, in which most are mesenchymal stem cells, with a few human pluripotent stem cell (hPSC)‐derived products and adult stem cell products. The indications of stem cell products were highly diversified, which include skin and subcutaneous disorders, graft versus host diseases, neuromuscular diseases, digestive disorders, pulmonary fibrosis, among others. The majority of INDs of gene therapy products are AAV products during 2018−2022, and 13 AAV products have been approved in clinical trials, which mainly aims to treat hemophilia and ophthalmology diseases. Different serotypes of are used in these AAV products, including AAV2, AAV9 and other new serotypes. For tissue‐engineered products, a number of products have been discussed in pre‐IND meetings, including 3D‐bioprinting blood vessels, human corneal endothelium and other types of tissue products, and some products are approved in clinical trials.

## 4 CHALLENGES AND PERSPECTIVES

Overall the regulatory reforms exerted profound influence on the innovation of cellular and gene therapy products in China.^21^ While the data show that China's ATMP industry is lagging behind in terms of the innovative targets and the coverage of indications. The increase of ATMP application in treating serious diseases could provide more therapeutic strategy and contribute to meeting the unmet clinical need.

Many ATMPs are translated from novel advances in still‐maturing fields of basic sciences, posing diverse challenges for development, including limited characterization, risk management in clinical trial and complex comparability study, such as individual donor tissue collection, multiple manufacturing sites, innovative device and materials, immune‐deficient animal model, and limited clinical subjects. Meanwhile, the heterogeneity of ATMPs is obvious, which arises from the variability in donor tissues, manufacturing processes and analytical methods, etc. And, limited production capacity, expensive price, and other factors prevent ATMPs from large‐scale production and widely commercial use.^22^

From CMC perspective, phase‐appropriate requirements for production of gene manipulation tools and other raw materials are important, while somehow difficult to articulate. Process changes inevitably happen in clinical trial and commercial stages, and the design and implementation of comparability study is crucial. Among all types of ATMPs, stem cell products are remarkably complex and varied in their manufacturing processes including methods for cell culture, induction of differentiation, cell storage and engraftment.^23^, ^24^ Generally it is difficult to assess whether prechange process and postchange process are analytically comparable, and nonclinical even clinical studies are needed in certain occasions.^25^ Therefore, in‐depth analysis and discussions are needed to share regulatory considerations on CMC, nonclinical and clinical experience of comparability assessments and ultimately achieve convergence.^26^

From the pre‐clinical aspect, there is no scientific consensus on rational animal models to test cellular products. Commonly, the challenges arise when we evaluate the potential tumorigenicity after cell product transplantation due to the difficulties in detecting the residual proliferative cells and the lack of proper animal models.^27^ In terms of the clinical trial, the use of history study data (e.g. Investigator‐Initiated Clinical Trial data) and endpoint selection strategy are difficult to justify. The outcomes of the clinical trials of ATMPs also bear uncertainty partly due to the disparities among patients. Therefore, it is challenging for the drug administrative agencies to perform the benefit‐risk analysis and review the ATMPs, which may be controversial in terms of safety and efficacy.^28^

Sound principles are required to regulate such types of complex products in a science‐based and risk‐based manner. For example, for the clinical use of genetically modified organisms, the environmental risk assessment is crucial in each application to prevent the potential harm to the environment and population.^29^ Regarding the genetically engineered cellular products, attentions should be paid to safety risks such as insertional mutations and activation of oncogenes. Tumorigenic/carcinogenic research can be designed based on the risk assessment results, which is typically more important for hPSC‐derived products with higher risky of tumor formation. Traditionally AAV is considered as nonintegrative vectors, while sequencing data suggest the potential integration capability of AAV in certain cells, which may impact the safety of AAV products.^30^ So, long‐term surveillance is still necessary to monitor safety and efficacy in AAV clinical trials.

To meet the continuous increasing medical need and foster the development of ATMPs, it is prior to establish the regulatory framework of ATMPs and to safely accelerate clinical applications.^31^ It is encouraged to use the expedited regulatory programs so as to make the review process more efficient for ATMPs, especially for those highly innovative products from small companies. And the agency will refashion the regulatory tools to establish a specific framework of ATMPs. In 2019, the National Drug Regulation Science Program of China has initiated and boosted the issuance of ATMP regulatory guidelines. According to the schedule, the Program will continuously support the high‐quality regulation of stem cell therapies and gene therapies to accomplish more targets in the following years (https://www.nmpa.gov.cn/directory/web/nmpa/yaowen/ypjgyw/20210628171415103.html). As a result, large numbers of technical guidelines were drafted and successfully issued in recent years. And, ATMPs of high‐quality and better efficacy increase significantly. Scientific judgment and cross‐discipline collaborations are encouraged to fulfill the true innovation and promote more ATMPs toward the process of market authorization in China. Consolidated efforts are required to strengthen the regulatory framework, including the update of guidelines, highly effective communications with stakeholders, fostering a partnership with international regulatory agencies to achieve convergence.^32^, ^33^, ^34^

## AUTHOR CONTRIBUTIONS

J.L. analyzed the data and wrote the manuscript. L.X. collected the data and revised the manuscript. W.W. proposed the topic and revised the manuscript. W.H. conceived and revised the manuscript. All authors read and approved the final manuscript.

## CONFLICT OF INTEREST STATEMENT

The authors declare no conflict of interests.

## FUNDING INFORMATION

The authors received no specific funding for this work.

## Data Availability

The data analyzed can be found in the database of NMPA's Registration and Information Disclosure Platform for Drug (https://www.nmpa.gov.cn/yaopin/index.html) and Center for Drug Evaluation (CDE) (http://www.cde.org.cn/).
